# Pre-Exascale Computing of Protein–Ligand Binding Free Energies with Open Source Software for Drug Design

**DOI:** 10.1021/acs.jcim.1c01445

**Published:** 2022-02-22

**Authors:** Vytautas Gapsys, David F. Hahn, Gary Tresadern, David L. Mobley, Markus Rampp, Bert L. de Groot

**Affiliations:** † Computational Biomolecular Dynamics Group, Max-Planck Institute for Biophysical Chemistry, Am Fassberg 11, 37077 Göttingen, Germany; ‡ Computational Chemistry, Janssen Research and Development, Janssen Pharmaceutica N. V., Turnhoutseweg 30, 2340 Beerse, Belgium; § Department of Pharmaceutical Sciences, University of California, Irvine, California 92697, United States; ∥ Max-Planck Computing and Data Facility, Giessenbachstrasse 2, 85748 Garching, Germany

## Abstract

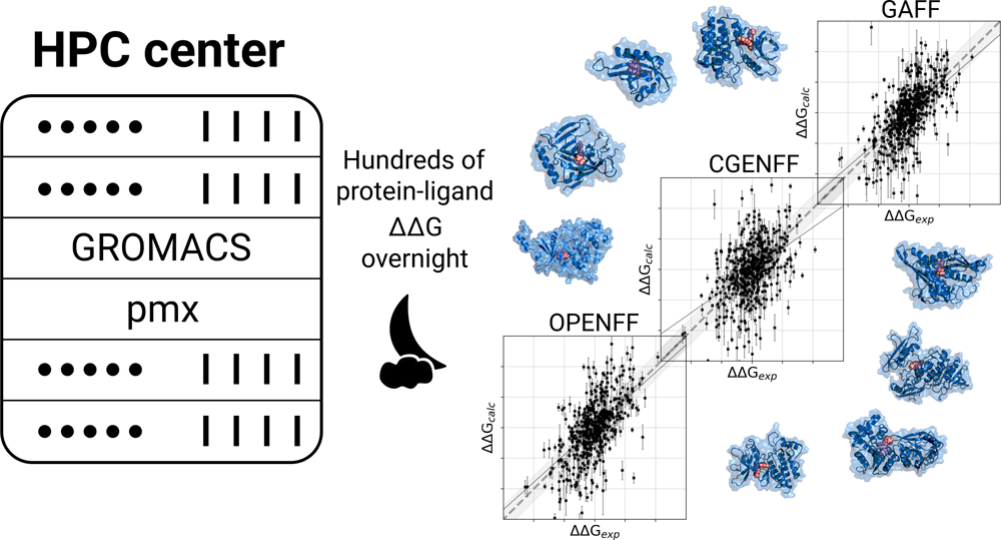

Nowadays, drug design projects benefit from highly accurate protein–ligand binding free energy predictions based on molecular dynamics simulations. While such calculations have been computationally expensive in the past, we now demonstrate that workflows built on open source software packages can efficiently leverage pre-exascale computing resources to screen hundreds of compounds in a matter of days. We report our results of free energy calculations on a large set of pharmaceutically relevant targets assembled to reflect industrial drug discovery projects.

## Introduction

Over the past decade, molecular dynamics-based alchemical free energy calculations have become widely adopted for assessing ligand–protein affinity changes upon protein mutation^1−4^ or, even more frequently, upon ligand modification.^5−9^ The methodology has become widely used in both the academic environment and pharmaceutical industry, where the computational predictions often aid and may even guide drug design efforts.

To facilitate such calculations, commercial^5,9,10^ and free^6,8^ solutions have been developed. The achieved accuracy and computational efficiency of these methods depend on a number of aspects, for example, force field, simulation engine, hybrid ligand structure/topology generation, and more. Therefore, it has become essential to evaluate the method performance on large benchmark sets comprising multiple diverse protein–ligand complexes. One such data set compiled by Wang et al.^5^ has readily become a standard in the community. This benchmark set was later extended by including additional protein–ligand systems.^8^ Another particularly useful contribution has been brought by Schindler et al;^7^ here, the authors presented a collection of publicly available data sets curated to represent protein–ligand systems investigated prospectively in Merck KGaA. The benchmark set is of special interest as it is tailored to reflect real-world application cases that are encountered in the pharmaceutical industry.

In our earlier work, we have used benchmark data sets collected from the literature to explore what prediction accuracies are achievable with the open source software and force fields.^8^ Following up on the latter investigation, we have now aimed to probe how quickly a large collection of benchmark systems collected by Schindler et al.^7^ could be evaluated when relying on the free open source software. Answering this question also demonstrates how drug development projects could be sped up by mere access to sufficient computational resources. For that we used Max Planck Society’s HPC Supercomputer “Raven” for three days to compute the whole data set with three different force fields, in total, performing ∼1.6 million molecular dynamics simulations, thus highlighting the scalability of the nonequilibrium free energy calculation protocol. As a result, we demonstrate that pre-exascale resources readily paved the way for large-scale and state-of-the-art molecular dynamics-based computational drug discovery projects to be run “overnight”, in contrast to months required on current smaller-scale or shared resources.

## Setup

**Figure 1:**
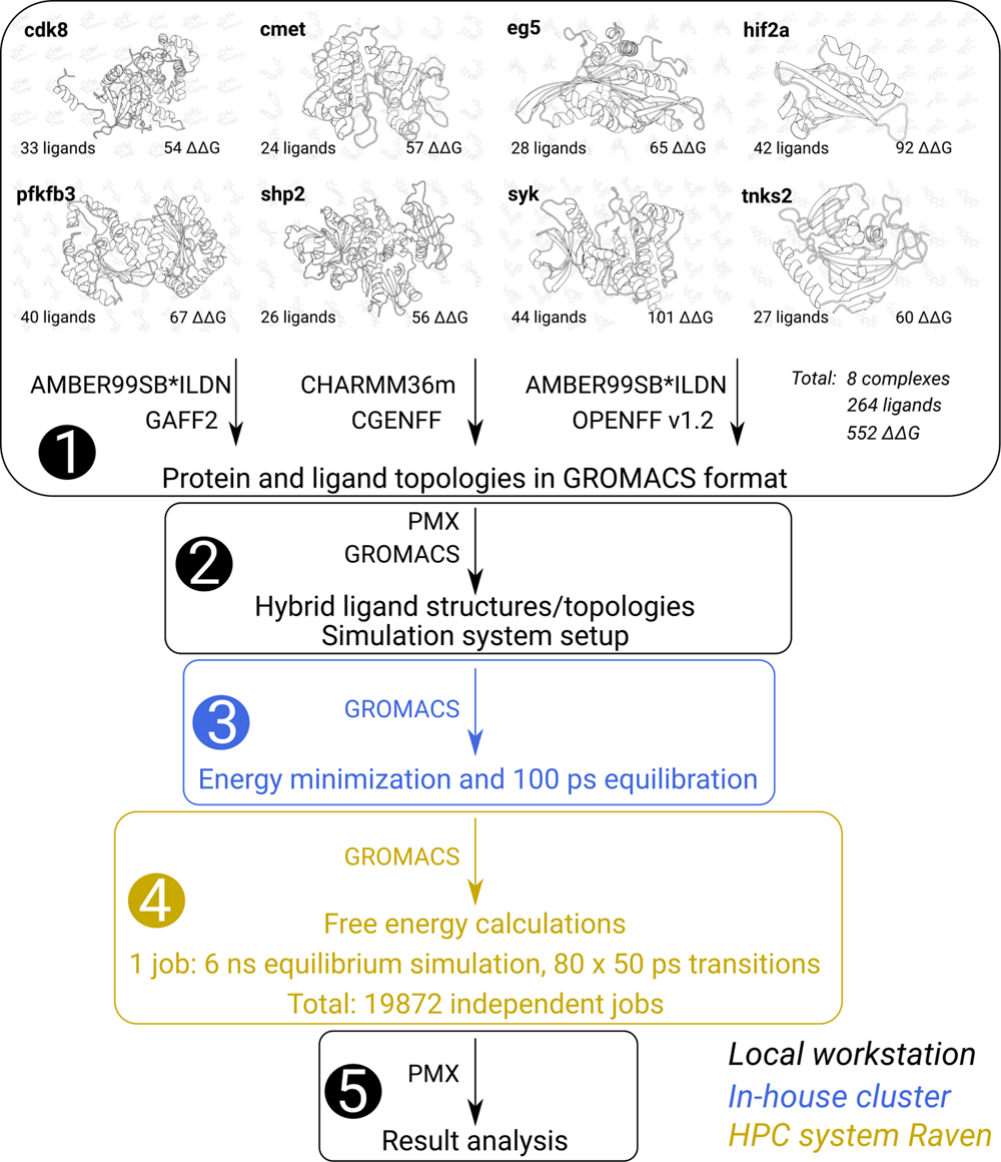
Workflow for the simulation procedure. (1) We start with the protein–ligand complexes prepared and made public by Schindler et al.^7^ The protein topologies are prepared in AMBER99SB*ILDN and CHARMM36m force fields, while for the ligands we used GAFF 2.11, CGENFF 3.0.1, and OpenFF 1.2.0 force fields. (2) Hybrid ligand structures and topologies for alchemical calculations are created with the pmx software, and further, the systems are assembled and prepared for the simulations with GROMACS. (3) Energy minimization and a brief 100 ps equilibration was performed on an in-house cluster. For the further automation of the workflow, this procedure could be merged with the following step and run in an HPC facility. (4) The main step where the simulations were performed on the Raven Supercomputer. We were able to make use of the trivial parallelization of the calculations by dividing the whole set into individual jobs as detailed in the text. (5) After finalizing the simulations on the HPC machine, the data were retrieved and analyzed locally.

The overall project workflow is summarized in Figure 1. We initialize the procedure with the protein–ligand complexes provided with the publication of the benchmark set assembled in Merck KGaA.^7^ This step of system assembly and cleaning, followed by the careful modeling of ligands, is highly important. Introducing ligand poses that do not reliably reflect actual ligand binding preferences would have severe consequences on the final free energy estimate accuracy. There are also numerous decisions required at this step: protein and ligand protonation states; protein starting structure selection; if needed, reconstruction of missing atoms and residues, and various additional aspects, many of which are summarized in the recent best practice guide.^11^ In the current work, we started with this step readily accomplished by Schindler et al.^7^ and continued our procedure with the topology generation.

This way, in the first step, for each of the considered complexes, we created GROMACS^12^ compatible topologies for various force fields. Proteins were represented by means of AMBER99SB*ILDN^13−15^ and CHARMM36m.^16^ For this, we employed the standard GROMACS topology generation tools. To parametrize the ligands, we chose three different force fields: GAFF^17^ version 2.11 topologies were created with the antechamber^18^ and ACPYPE^19^ software. MATCH^20^ was used for assigning the CGENFF v3.0.1 parameters. We have also included a version v1.2.0 Parsley of the recently developed OpenFF^21^ force field. The OpenFF topologies were generated using the OpenFF toolkit^22^ and converted to GROMACS topologies using ParmEd.^23^ For the further simulations, GAFF and OpenFF were combined with the AMBER99SB*ILDN protein force field, while CGENFF was used in combination with CHARMM36m.

As at this step we did not employ high level quantum chemical calculations (AM1-bcc charges^24^ for GAFF and OpenFF; MATCH assigned charges based on bond charge increment rules for CGENFF), the step only takes up to several minutes per ligand. If a more elaborate parametrization is desired, it may become more time efficient to perform the computationally costly QM calculations on an in-house cluster or at an HPC facility.

Afterward, in the second step of the procedure, we created hybrid structures and topologies for the ligand pairs using the pmx^25^ software. To enable equivalent comparison with the previously published results, we have chosen to evaluate free energy differences between the same ligand pairs as reported by Schindler et al.^7^ This step is not computationally demanding and can be performed sequentially in a matter of minutes or hours even for a large set of perturbations. The generated hybrid structures were then assembled together with the protein structures, and a standard GROMACS procedure of system solvation and addition of salt was performed.

Up to this point, the prepared systems are agnostic to the specific free energy protocol; i.e., they can be used for the free energy perturbation (FEP); discrete, slow growth, or nonequilibrium thermodynamic integration; or any other alchemical protocol of interest. Here, based on our experience in a previous investigation,^8^ we have chosen to use the nonequilibrium free energy calculation procedure. To briefly outline the procedure, we equilibrate the system in its two physical end states representing the two ligands that are perturbed into one another. Subsequently, from the trajectories generated in equilibrium (6 ns per run), we extract 80 snapshots and start a quick 50 ps transition from one physical state to the other. The whole procedure is performed for two branches of the thermodynamic cycle: perturbation in water and in a protein–ligand complex. Also, to obtain a reliable uncertainty estimate, each ΔΔ*G* calculation was performed using three independent replicates.

As a preparatory step, we have performed an energy minimization and a brief equilibration of the system for 100 ps (step 3 in Figure 1) on an in-house cluster. In principle, this step could be merged with the following main calculation performed on the HPC Supercomputer. For the current project, however, we decided for an option of carrying out initial short simulations on an in-house computer cluster. This way, we ensured that the prepared systems were stable and ready to be transferred to the HPC Supercomputer Raven for the actual free energy calculations. Since in an everyday application this step would be a part of the next step (step 4 in Figure 1), its timing is of no particular importance, as it constitutes only a minor fraction of the full free energy computation.

The fourth step in Figure 1 is the main point of the computations in this letter highlighting the scaling capabilities for such calculations. While the GROMACS simulation engine itself offers high throughput in terms of generated trajectory time,^26^ the employed free energy calculation protocol further allows for trivial parallelization of the jobs. Overall, we could divide the whole scan into 19,872 independent jobs: 552 ΔΔ*G* calculations in three force fields for two thermodynamic branches (water, protein–ligand), each of which requires two simulations (one for forward and one for backward direction) and three independent replicas for each calculation. In total, ∼200 μs of a simulation trajectory was generated in this scan. The whole simulation was accomplished in approximately three days, leveraging resources allocated during the testing phase of the Max Planck Supercomputer Raven (interim) allowing one to simultaneously use 480 Intel Xeon Cascade Lake-AP nodes with 96 cores (192 threads) each.

The current division of simulations into separate jobs was dictated by the available resources and could be easily modified to match a specific HPC architecture. For example, having access to a particularly large computer facility one could further separate every short 50 ps transition into an individual job allowing one to run ∼1.6 million small jobs in parallel, thus further reducing the waiting time to prediction.

In the final step, the generated output was transferred from the HPC facility and analyzed on a local workstation by means of the pmx software. The accuracies of the predicted free energies were further explored by comparison to the experiment and previous calculations.

## Results: Calculation Accuracy

**Figure 2:**
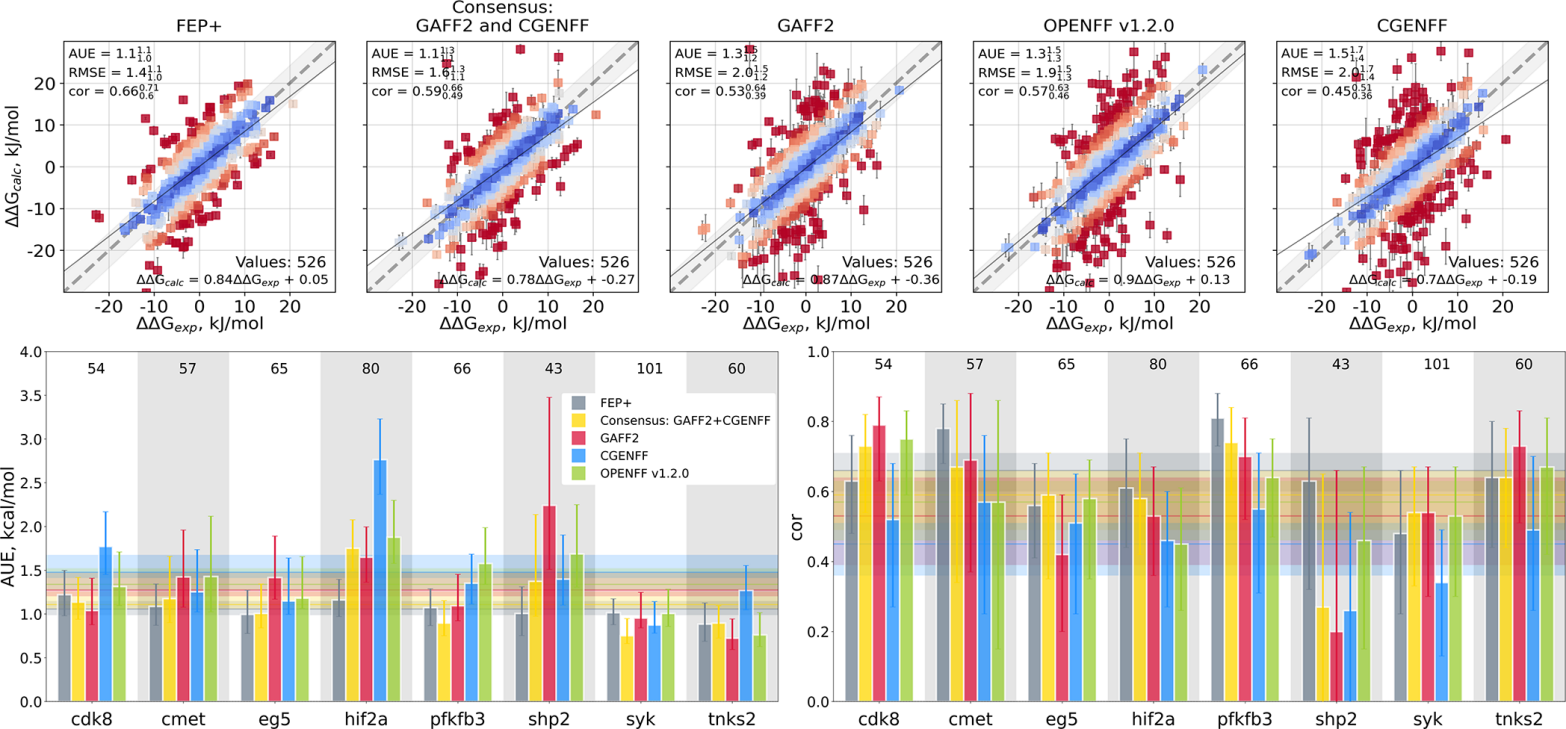
Comparison of the computed ΔΔ*G* values to the experimental measurements. (Top) Scatter plots of all the values that have been reported by Schindler et al.^7^ for the FEP+ 5 ns per window calculation protocol. In adherence with the best practice,^11^ the directions of the ΔΔ*G* edges have been retained exactly the same as in ref (7). The first panel reports FEP+ 5 ns results by Schindler et al., while the other panels present results from the current work. (Bottom) Average unsigned error (AUE) and Pearson correlation (cor) for each protein–ligand complex separately. The horizontal lines denote mean values. The numbers in the panels report the free energy differences calculated for each system. In the analysis, we consider 526 ΔΔ*G* estimates that had values reported for the FEP+ 5 ns protocol.

Overall, the calculation accuracy matches well our earlier observations for a different protein–ligand benchmark set.^8^ Relying on our earlier experience,^8^ we have constructed the consensus approach combining results from two different families of force fields: GAFF and CGENFF (we have not included OpenFF in the consensus, because its early 1.2.0 version mainly aims at reproducing the behavior of GAFF). In turn, this yields better accuracy in terms of agreement with the experimentally measured values than the force fields considered individually when comparing predicted ΔΔ*G* with experimental measurements (Figure 2, Figure S1). The consensus calculations (AUE 1.1_1.1_^1.3^ kcal/mol, Pearson correlation 0.59_0.49_^0.66^) also approach the performance of the commercial software FEP+ (AUE 1.1_1.0_^1.1^, Pearson correlation 0.66_0.6_^0.71^).

Individually, GAFF 2.11 and OpenFF achieved comparable accuracy and performed better than the CGENFF force field. It could not be excluded that the results obtained with the CGENFF force field could be further improved by employing a newer force field version, as currently we relied on an older parameter set (3.0.1). To probe sensitivity of the results to the force field version, we have performed calculations on the same set of systems by using bonded ligand parameters (bonds, angles, dihedrals) from the newer CGENFF 4.6 version. The nonbonded ligand parameters, as well as all the protein, water, and ion parameters, were retained the same as in the earlier simulations. It appears that this way an upgraded force field does not warrant higher prediction accuracy (Figure S2). Of course, this test does not mean that improving on force fields is a futile task, but rather it suggests that to see significant improvement larger modifications might be required, for example, improvements on atom type assignment for specific chemical groups or additional QM-based parametrizations.

Regarding the OpenFF force field, here we have benchmarked an early version (v1.2.0 Parsley) of the force field. At the time when the calculations were performed, this OpenFF version had not yet undergone Lennard-Jones parameter reparameterization. Recently, OpenFF v2.0 has been released, and some preliminary calculations indicate its improved accuracy in ΔΔ*G* predictions.^27^ Therefore, in the future, it would be interesting to probe how much the accuracy would improve by employing the updated force field versions.

In the bottom panels of Figure 2 (and Figure S1), we show the breakdown of the calculated ΔΔ*G* values by protein–ligand complex. The performance of the individual force fields depends on the system simulated and is often strongly influenced by large outliers; for example, the overall well-behaved GAFF force field shows a reduced accuracy for the shp2 complex mainly due to a number of poor predictions. The consensus approach often suppresses the largest deviations from the experimental measurements. Modeling of the initial ligand pose also plays an important role for the result accuracy. For example, for the cmet protein–ligand complex, Schindler et al. reported the results after probing several modeled poses (personal communication). In the current work, we used a single pose which in some cases was suboptimal for the cmet system, in turn yielding more outliers and lowering prediction accuracy.

It is also important to note that while we have computed all 552 ΔΔ*G* values for the ligand maps from the work by Schindler et al.,^7^ some entries were not present for the FEP+ calculation protocol using 5 ns simulations. In order to have a consistent comparison, we have retained 526 ΔΔ*G* estimates that had values reported for the FEP+ 5 ns protocol. We have also ensured that using the whole data set does not have a significant effect on the obtained accuracy (Figure S3).

**Figure 3:**
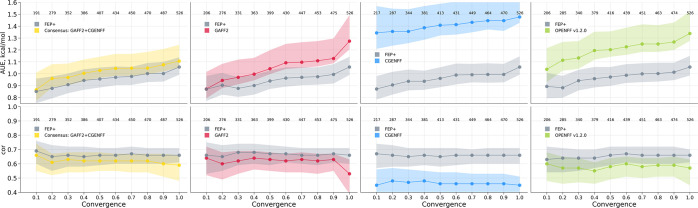
Prediction accuracy in terms of the average unsigned error (top) and Pearson correlation (bottom) with respect to convergence of the ΔΔ*G* estimates. For the convergence analysis, we used the measure derived by Hahn and Then,^28^ the application of which to the alchemical free energy calculations we have described previously.^29^ The measure is defined in the range [−1;1], where the values close to 0 denote well-converged estimates; thus, the smaller values on the *x*-axis denote better convergence. It is important to note that this measure only reports on the convergence of the estimator, but does not include information on the potential lack of sampling in the relevant phase space regions for either of the physical end states. The data points depicted in the figure were calculated by considering subsets of points below a corresponding convergence threshold; for example, for a convergence value of 0.8, only those data points were considered that had an average convergence in the protein–ligand branch of the thermodynamic cycle less than or equal to 0.8. The numbers in the panels indicate how many ΔΔ*G* values were considered given a corresponding convergence threshold. FEP+ results correspond to the Schindler et al.^7^ 5 ns simulation protocol calculated for the same ΔΔ*G* values that have been identified for a corresponding convergence threshold for the simulations in the current work. The FEP+ curves in the panels differ due to the fact that disparate ligand pairs are considered at varying convergence levels for different force fields.

An important question always accompanying molecular dynamics-based methods is whether the simulations are sufficiently converged. Schindler et al. extended their simulation by a factor of 4, thus reaching 20 ns sampling for each window; this resulted in a modest and statistically insignificant increase in prediction accuracy (Figure S4). Similarly, we have also probed whether better convergence would increase prediction accuracy in case of our calculations. To this end, we have selected cdk8 protein–ligand complexes and performed the transition simulations between the end states two times slower (100 ps per transition). Similarly to the observations with the increased sampling in FEP+ case, we observed only an insignificant increase in accuracy (Figure S5). This test, however, does not exclude the possibility that reaching a sufficient convergence (irrespective of the required sampling time), could further improve the prediction accuracy. In fact, we do observe a closer agreement with the experiment for those ΔΔ*G* estimates that are converged better (Figure 3).

In these cases of convergence assessment, we have mainly concentrated on the convergence of the free energy estimate itself. However, it is possible to obtain a well-converged estimate, yet if it reports on a free energy difference between states that do not match those observed in experiment, the prediction accuracy will be poor. An example of this situation is system eg5, where alternative loop conformations in the vicinity of the ligand binding site yield different ΔΔ*G* accuracies (Figure S6). Only given a sufficiently long sampling time, one might expect establishing reliable population ratios between largely different conformers.

As it was not the main aim of the current letter to investigate all the particular details of the predicted ΔΔ*G* values and their force field dependence, together with the manuscript we provide all the calculated data. We are further planning to incorporate the data generated in this scan into a larger benchmark study comprising protein–ligand complexes assembled from numerous benchmark sets (refs (5, 7, 8), and others) and comparing free energy predictions from multiple force fields and their different versions.

## Conclusions

In the current letter, we highlight that rapid high throughput sampling of protein–ligand binding affinities is readily achievable. Provided that sufficient computational resources are available, large scale alchemical protein–ligand binding free energy predictions can be efficiently run solely relying on the open source software in a routine fashion to guide drug discovery projects. Screening hundreds of derivatives of an initial hit or lead compound can be achieved in a matter of days while obtaining the high accuracy of alchemical free energy calculations. Our results show how the accuracy of prediction versus experiment differs with each force field for the same free energy calculation approach. It is expected that improvements in force field, such as newer versions of the OpenFF, can lead to even better accuracy, as shown to be the case with each newer iteration of OPLS when used with the same FEP+ approach. A consensus approach combining the results from multiple force fields generally additionally improves accuracy.

## Data and Software Availability

The calculations were performed with the publicly available free open source software. The calculated free energy values, ligand and protein structures, and topologies are available at https://github.com/deGrootLab/rel_ddG_MerckDataSet_JCIM.

## Supporting Information Available

Scatterplots of the experimental against calculated ΔΔ*G* values for all systems and force fields separately, prediction accuracy for the CGENFF 3.0.1 and 4.6 versions, comparison of the predicted ΔΔ*G* to experiment for all 552 values, prediction accuracy comparison including 20 ns FEP+ protocol, and assessment of the simulation length on the free energy prediction for the cdk8 system and two alternative conformers of the eg5 system (PDF)
