# Supplementary material for: Pre-Exascale Computing of Protein–Ligand Binding Free Energies with Open Source Software for Drug Design
Source: J Chem Inf Model. 2022 Feb 22;62(5):1172–7. doi: 10.1021/acs.jcim.1c01445 (PMC8924919; doi:10.1021/acs.jcim.1c01445)
Supplement: Supplementary file 1 — ci1c01445_si_001.pdf [file ci1c01445_si_001.pdf]

# Supporting Information: Pre-exascale computing of protein-ligand binding free energies with open source software for drug design

Vytautas Gapsys,[*,†] David F. Hahn,[‡] Gary Tresadern,[‡] David L. Mobley,[¶] Markus Rampp,[§] and Bert L. de Groot[*,†]

†*Computational Biomolecular Dynamics Group, Max-Planck Institute for Biophysical Chemistry, Am Fassberg 11, 37077, Göttingen, Germany*

‡*Computational Chemistry, Janssen Research & Development, Janssen Pharmaceutica N. V., Turnhoutseweg 30, 2340, Beerse, Belgium*

¶*Department of Pharmaceutical Sciences, University of California, Irvine, CA 92697, USA*

§*Max-Planck Computing and Data Facility, Giessenbachstrasse 2, 85748, Garching, Germany*

E-mail: vgapsys@gwdg.de; bgroot@gwdg.de

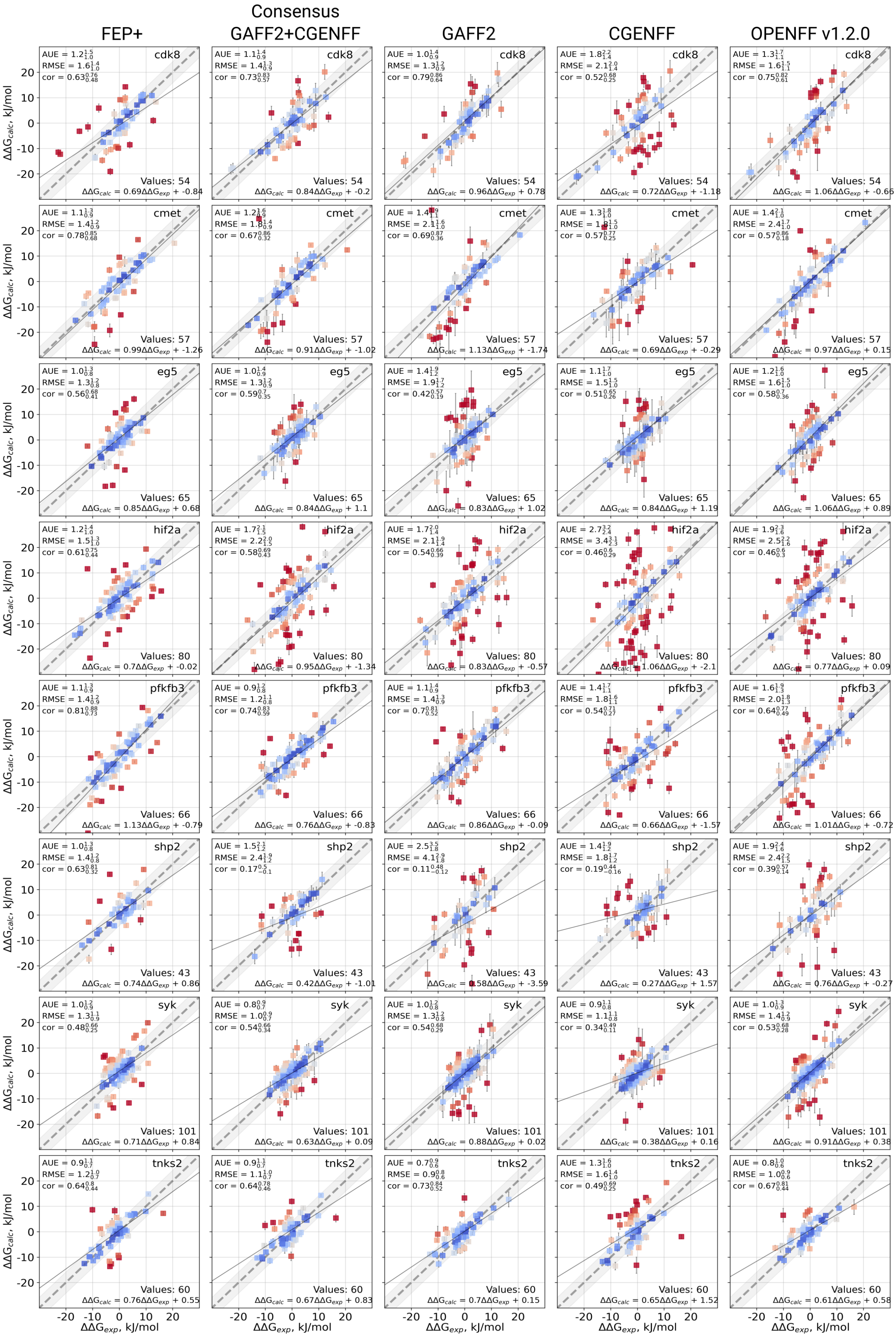


**Figure S1:** Scatterplots for all calculated ΔΔG values against the experimental measurements. The first column presents FEP+ data for 5 ns per window simulation protocol from Schindler et al[1]. The other columns contain data from the calculations in the current work.

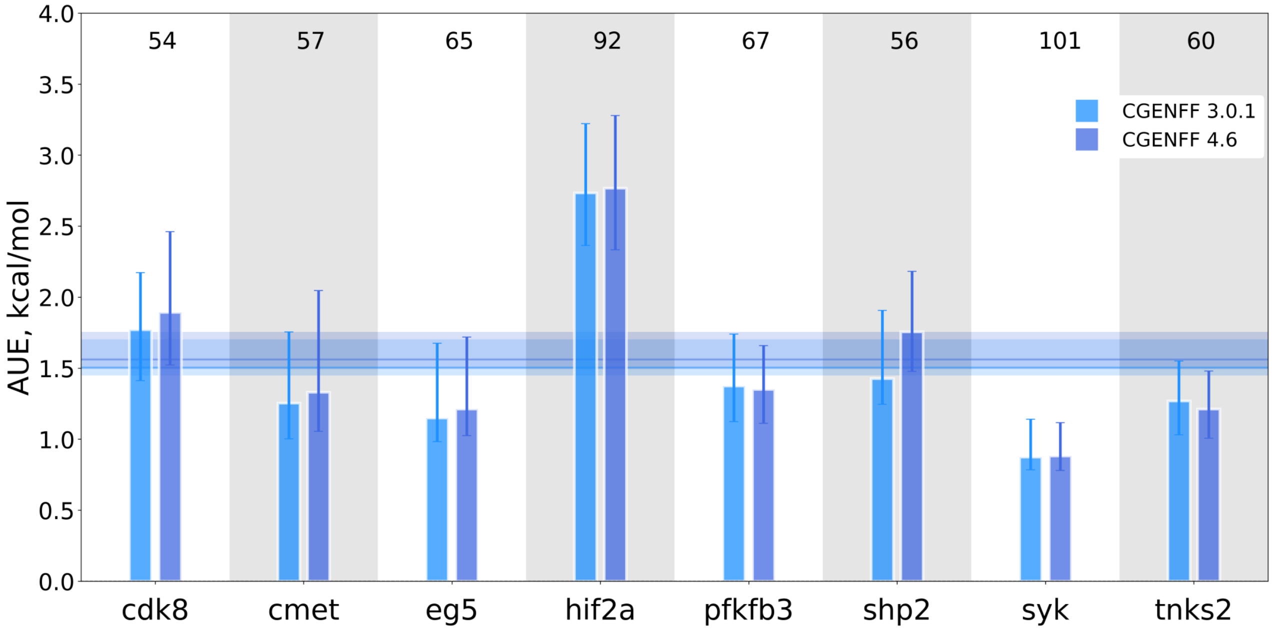


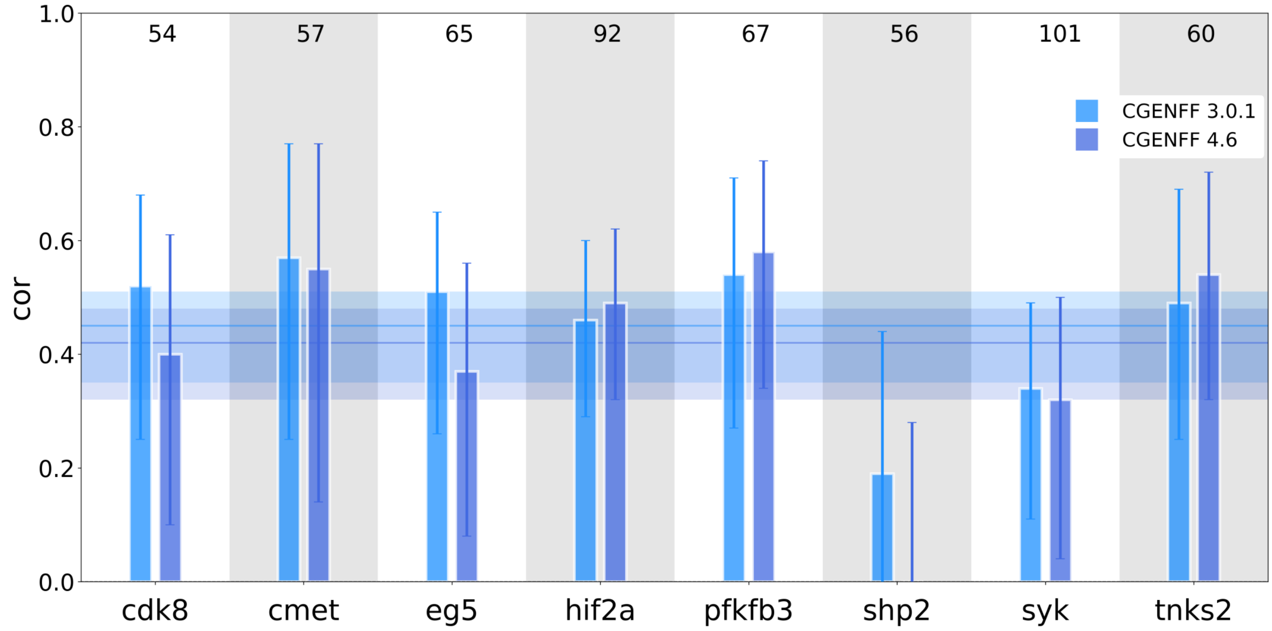


**Figure S2:** Comparison of the ΔΔG prediction accuracy for the CGENFF 3.0.1 and 4.6 versions. Only the ligand bonded parameters differ between the two sets of calculations, while the non-bonded parameters, as well as all the protein, water, ion parameters are retained. The comparisons are depicted in terms of the average unsigned error (top panel) and Pearson correlation (bottom panel).

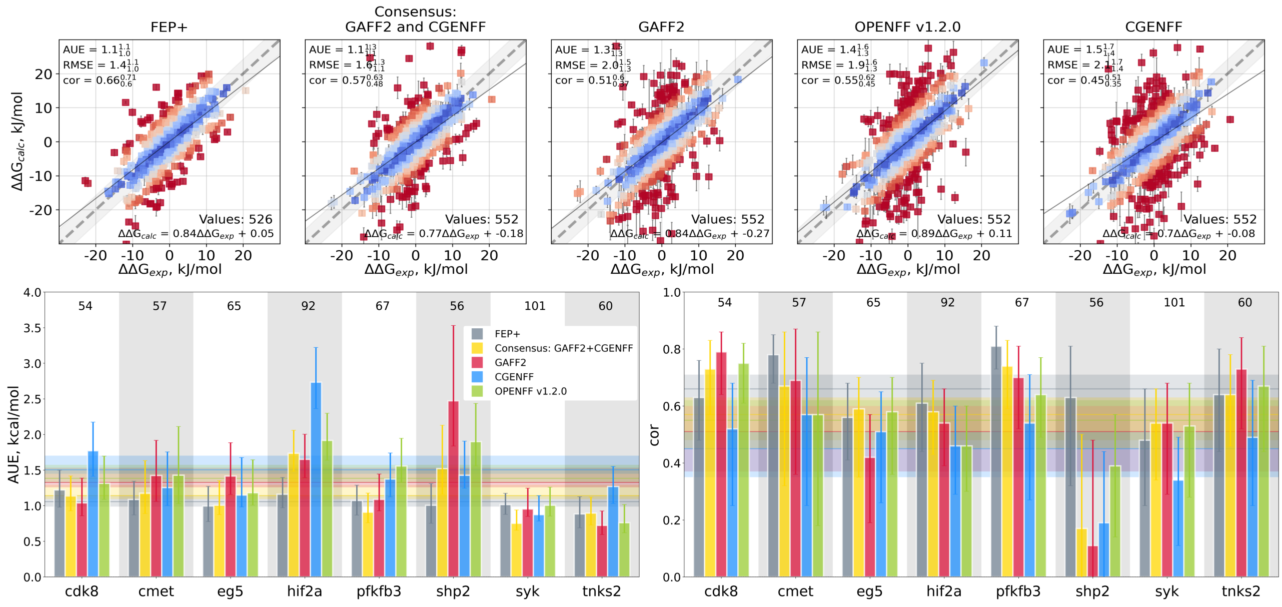


**Figure S3:** Comparison of the computed ΔΔG values to the experimental measurements: all 552 values are considered for the calculations performed in this work. Top row: the first panel reports FEP+ 5 ns protocol results by Schindler et al, while the other panels present results from the current work. Bottom row: average unsigned error (AUE) and Pearson correlation (cor) for each protein-ligand complex separately. The horizontal lines denote mean values. The numbers in the panels report on the free energy differences calculated for each system.

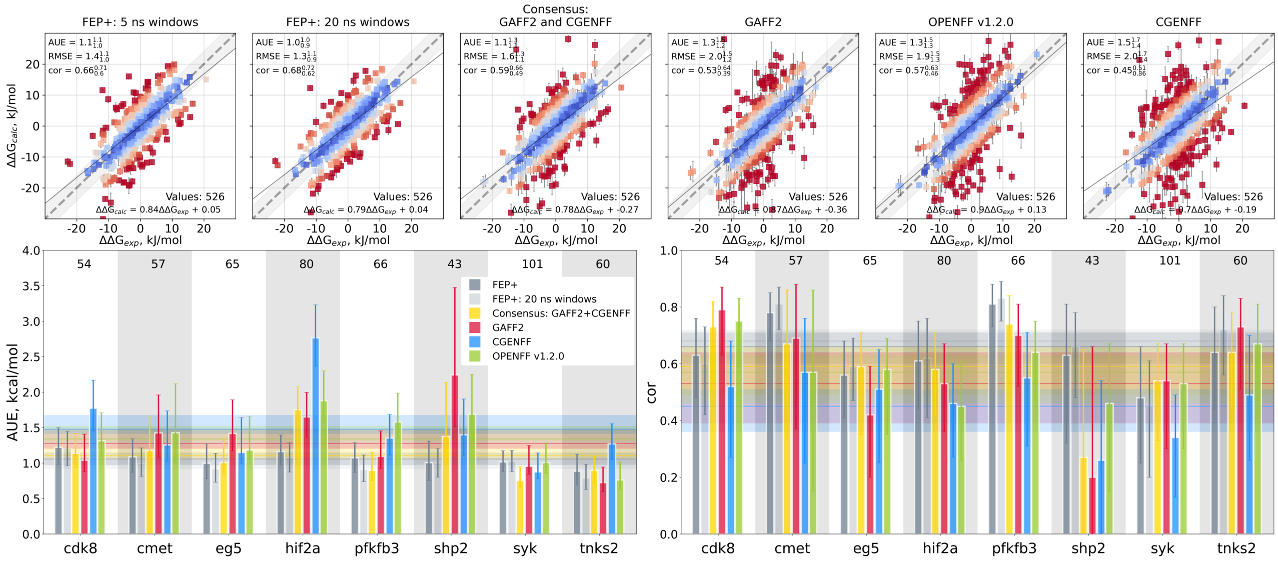

**Figure S4:** Comparison of the computed ΔΔG values to the experimental measurements: including 5 ns and 20 ns FEP+ protocols. Top row: scatter plots of all the values that have been reported by Schindler et al[1] for the FEP+ 5 ns per window protocol. The first panel reports FEP+ 20 ns per window simulation results by Schindler et al, the second panel reports FEP+ 5 ns simulation results by Schindler et al, the other panels present results from the current work. Bottom row: average unsigned error (AUE) and Pearson correlation (cor) for each protein-ligand complex separately. The horizontal lines denote mean values. The numbers in the panels report on the free energy differences calculated for each system.

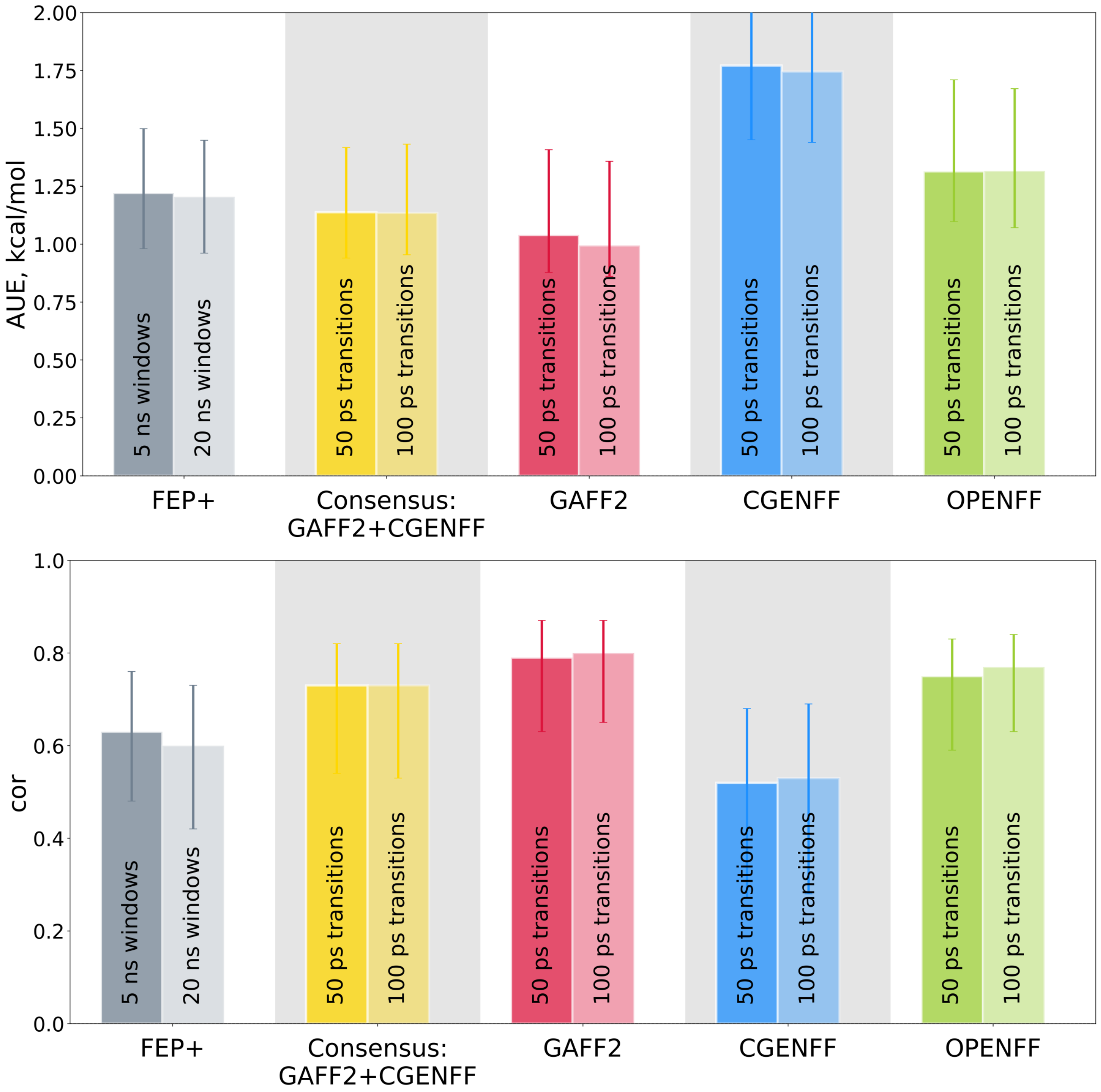


**Figure S5:** Comparison of the ΔΔG prediction accuracy for the cdk8 system following simulation protocols of different length. For FEP+, the 5 ns and 20 ns per window simulation results from Schindler et al[1] are shown. For the other force fields we report calculations with the shorter (50 ps) and longer (100 ps) transitions between the physical end states. The longer simulations do not significantly affect the prediction accuracy. The comparisons are depicted in terms of the average unsigned error (top panel) and Pearson correlation (bottom panel).

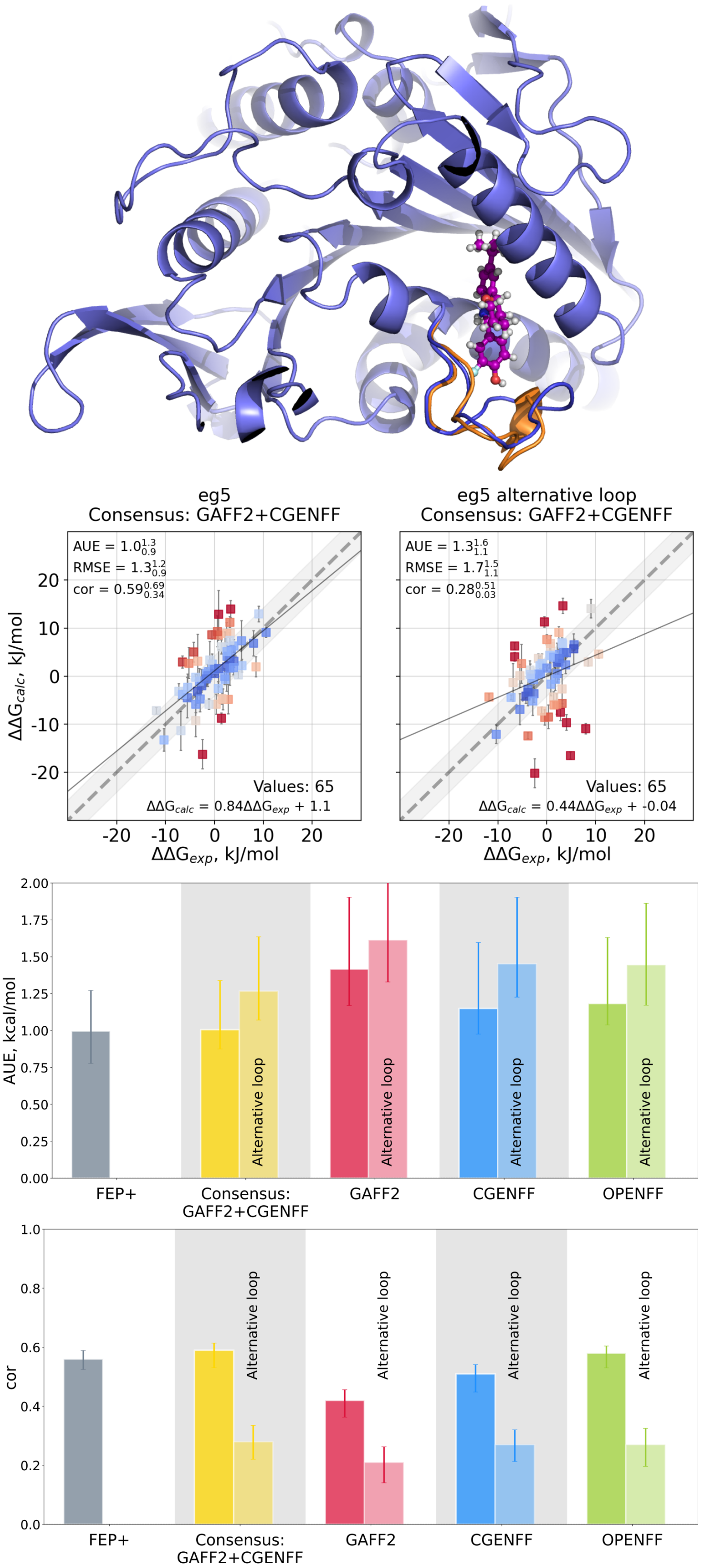


**Figure S6:** Prediction accuracy for the two alternative conformers of the eg5 system. In the top row two structures of the eg5 protein-ligand complex are depicted, where an alternative loop conformation is colored in orange. The scatter plots (second row), as well as the average unsigned error (third row) and Pearson correlation (fourth row) analyses illustrate reduced accuracy for the conformer containing the alternative loop state.

## References

(1) Schindler, C. E. M.; Baumann, H.; Blum, A.; Böse, D.; Buchstaller, H.-P.; Burgdorf, L.; Cappel, D.; Chekler, E.; Czodrowski, P.; Dorsch, D.; Eguida, M. K. I.; Follows, B.; Fuchß, T.; Grädler, U.; Gunera, J.; Johnson, T.; Jorand Lebrun, C.; Karra, S.; Klein, M.; Knehans, T.; Koetzner, L.; Krier, M.; Leiendecker, M.; Leuthner, B.; Li, L.; Mochalkin, I.; Musil, D.; Neagu, C.; Rippmann, F.; Schiemann, K.; Schulz, R.; Steinbrecher, T.; Tanzer, E.-M.; Unzue Lopez, A.; Viacava Follis, A.; Wegener, A.; Kuhn, D. Large-Scale Assessment of Binding Free Energy Calculations in Active Drug Discovery Projects. *J. Chem. Inf. Model.* **2020**, *60*, 5457–5474.
